# Isolation and characterization of *Burkholderia fungorum* Gan-35 with the outstanding ammonia nitrogen-degrading ability from the tailings of rare-earth-element mines in southern Jiangxi, China

**DOI:** 10.1186/s13568-017-0434-x

**Published:** 2017-06-26

**Authors:** Ai-Juan Feng, Xi Xiao, Cong-Cong Ye, Xiao-Ming Xu, Qing Zhu, Jian-Ping Yuan, Yue-Hui Hong, Jiang-Hai Wang

**Affiliations:** 1 School of Life Sciences, Sun Yat-Sen University, Guangzhou, 510275 People’s Republic of China; 2 Guangdong Provincial Key Laboratory of Marine Resources and Coastal Engineering/South China Sea Bioresource Exploitation and Utilization Collaborative Innovation Center, School of Marine Sciences, Sun Yat-Sen University, Guangzhou, 510006 People’s Republic of China

**Keywords:** Ammonia nitrogen, Bioremediation, *Burkholderia*, Rare-earth-element mine

## Abstract

The exploitation of rare-earth-element (REE) mines has resulted in severe ammonia nitrogen pollution and induced hazards to environments and human health. Screening microorganisms with the ammonia nitrogen-degrading ability provides a basis for bioremediation of ammonia nitrogen-polluted environments. In this study, a bacterium with the outstanding ammonia nitrogen-degrading capability was isolated from the tailings of REE mines in southern Jiangxi Province, China. This strain was identified as *Burkholderia fungorum* Gan-35 according to phenotypic and phylogenetic analyses. The optimal conditions for ammonia–nitrogen degradation by strain Gan-35 were determined as follows: pH value, 7.5; inoculum dose, 10%; incubation time, 44 h; temperature, 30 °C; and C/N ratio, 15:1. Strain Gan-35 degraded 68.6% of ammonia nitrogen under the optimized conditions. *Nepeta cataria* grew obviously better in the ammonia nitrogen-polluted soil with strain Gan-35 than that without inoculation, and the decrease in ammonia–nitrogen contents of the former was also more obvious than the latter. Besides, strain Gan-35 exhibited the tolerance to high salinities. In summary, strain Gan-35 harbors the ability of both ammonia–nitrogen degradation at high concentrations and promoting plant growth. This work has reported a *Burkholderia* strain with the ammonia nitrogen-degrading capability for the first time and is also the first study on the isolation of a bacterium with the ammonia nitrogen-degrading ability from the tailings of REE mines. The results are useful for developing an effective method for microbial remediation of the ammonia nitrogen-polluted tailings of REE mines.

**Electronic supplementary material:**

The online version of this article (doi:10.1186/s13568-017-0434-x) contains supplementary material, which is available to authorized users.

## Background

Rare earth elements (REEs) have wide applications and are considered as the industrial gold due to their unique optical, magnetic, and catalytic properties (Cornell 1993). Currently, China supplies over 90% of the REEs-related products to the global market, and two-thirds of the products are produced in southern Jiangxi Province (Information Office of the State Council of China 2010). Among the REE mines, the ion-absorbing middle-heavy REE deposit occupies an important position in the world market (Information Office of the State Council of China 2010). REEs in this deposit primarily occur in the weathered layer of granites and are generally adsorbed in soils/sediments in the form of ions (Bao and Zhao 2008). So far, the advanced in situ leaching method has been extensively adopted to separate and extract ion-adsorbed REEs in southern Jiangxi Province, China (Wen et al. 2013). This is an effective method for the REE exploitation. However, it depends on the injection of chemicals, such as ammonium sulfate or ammonium bicarbonate, into the soils/sediments to extract REEs. The tailings and waste water resulting from the exploitation contains high concentrations of ammonia nitrogen (NH_4_^+^-N), which have caused severe negative impacts on local ecosystems and human health (Åström 2001). For instance, the NH_4_^+^-N pollution in the tailings of REE mines has resulted in soil degradation, forest destruction, and threat to life (Gao and Zhou 2011). The carcinogenic effect may be induced when NH_4_^+^-N in the polluted drinking water was transformed into nitrite nitrogen (NO_2_^−^-N). Therefore, it is urgent and necessary to remediate the NH_4_^+^-N-polluted tailings in REE mines for realizing a sustainable development.

Recently, diverse methods have been proposed for environmental remediation. Besides high cost, the physical and chemical methods can not thoroughly eliminate pollutants and may result in secondary pollution. Thus, they are usually used for emergent environmental restoration (Xue et al. 2015). Bioremediation has become one of the most reliable strategies for completely eliminating pollutants without secondary pollution. Recent researches on the tailings of REE mines has focused on the REE risk in soils and vegetables to human health (Hao et al. 2016). To our knowledge, however, studies on the bioremediation of the tailings of REE mines are rare, which is hindering the realization of pollution reduction and anticipated ecological balance in these areas. Microbial remediation is one of bioremediation methods and has been regarded as a cost-effective and eco-friendly strategy for eliminating pollutants (Al-Mailem et al. 2014; Dellagnezze et al. 2014; Hassanshahian et al. 2012). Screening microorganisms with the NH_4_^+^-N-degrading ability in the tailings of REE mines is undoubtedly important for performing microbial remediation in these areas. However, up to date, no report on the microorganisms with the NH_4_^+^-N-degrading ability is present in these areas.

The aim of this study is to isolate and characterize a bacterium with the outstanding NH_4_^+^-N-degrading capability from the tailings of REE mines in southern Jiangxi Province, China. The results may contribute to developing an effective method for the microbial remediation of NH_4_^+^-N-polluted environments, in particular the tailings of REE mines.

## Methods

### Sampling

**Fig. 1:**
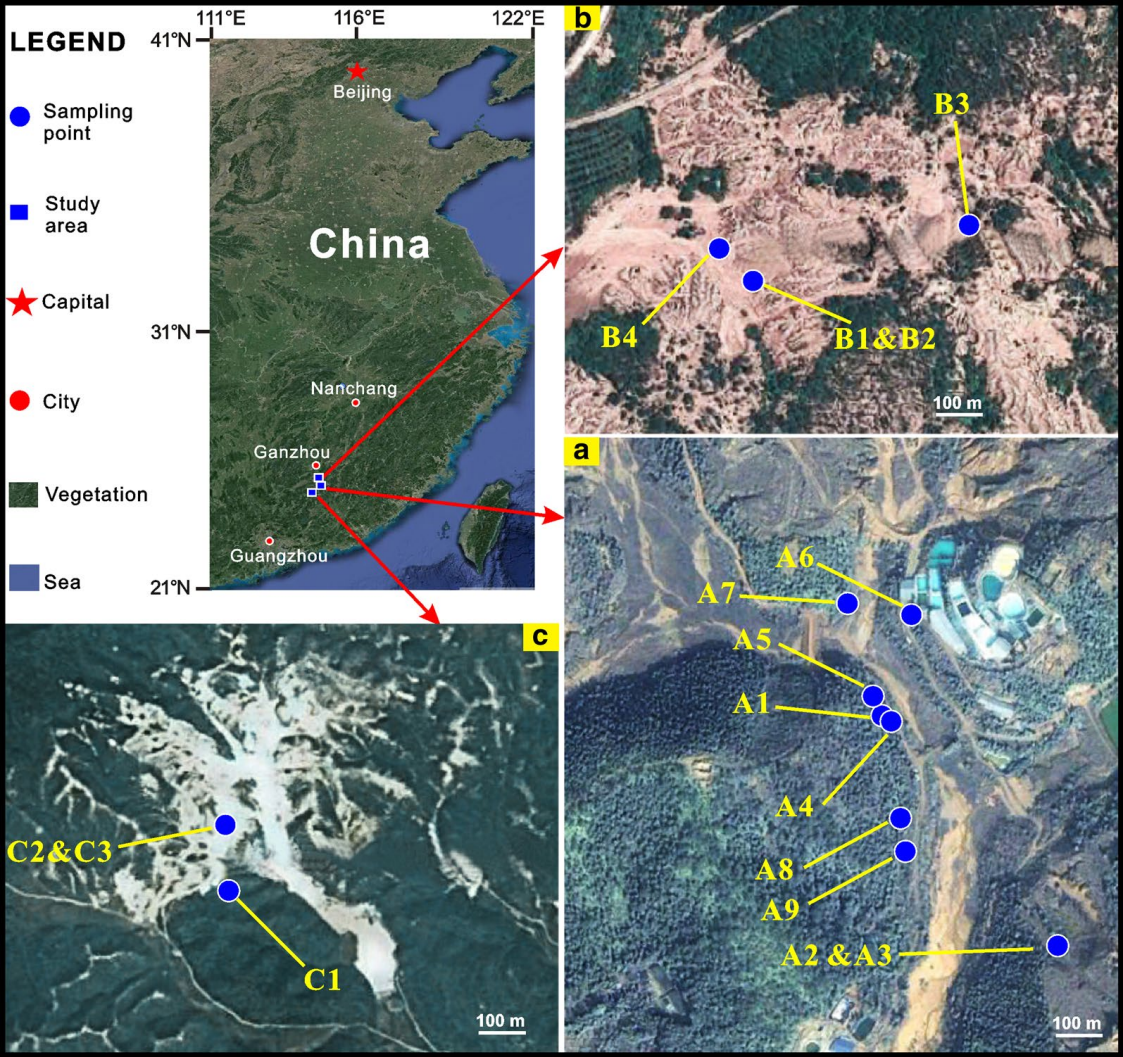
Remote sensing images of three rare-earth-element mines in southern Jiangxi, China. The geomorphologic features and 16 sampling sites (*blue circles*) are shown in the figure

Sixteen tailing samples were obtained from three REE mines with the severe NH_4_^+^-N pollution in southern Jiangxi Province, China. The sampling sites were randomly selected near the exploitation areas of the REE mines (Fig. 1). The samples were excavated from the depth of 10–15 cm in the tailings. Then, the samples were transferred into sterile bags, sealed and kept in a nitrogen canister. After being taken back to the laboratory, the samples were stored at −20 °C until being used for analysis.

### Culture media

The enrichment medium (pH 7.2–7.4) was composed of (g/L): glucose, 5; (NH_4_)_2_SO_4_, 5; NaCl, 2; FeSO_4_·7H_2_O, 0.4; K_2_HPO_4_, 1; and MgSO_4_·7H_2_O, 0.5. The Luria–Bertani (LB) liquid medium consisted of (g/L): yeast extract, 5; tryptone, 10; and NaCl, 10. The LB agar medium contained (g/L): yeast extract, 5; agar, 20; NaCl, 10; and tryptone, 10. The screening medium (pH 7.2–7.4) was composed of (g/L): glucose, 5; (NH_4_)_2_SO_4_, 5; NaCl, 1; K_2_HPO_4_, 0.5; and MgSO_4_·7H_2_O, 0.25. All the culture media were prepared using deionized water and were autoclaved for 30 min before use.

### Analysis of the contents of NH_4_^+^-N, NO_3_^−^-N and NO_2_^−^-N in the tailings

The concentrations of NH_4_^+^-N in the tailings were measured by spectrophotometry using the Auto Analyser 3 System (Bran + Luebbe, Germany). Prior to analysis, 25 g of the samples were mixed with 100 mL of deionized water, respectively. The concentrations of NH_4_^+^-N were measured using hydrazine sulphate (Kearns 1968) as a color marker. The obtained results were corrected for the amount of the samples and expressed as milligram per kilogram of the tailings. The contents of NO_3_^−^-N were measured according to the international method (Liang et al. 2012), which was based on the absorbance of NO_3_^−^ at 220 nm. The contents of NO_2_^−^-N were determined by measuring the absorbance of NO_2_^−^-N solution at 540 nm according to the instructions of an international standard method (Shi and Chao 2014). This method is based on the following principle: (i) NO_2_^−^ reacts with 4-aminobenzenesul fonamide under the condition of pH 1.8, resulting in the production of diazonium salt; (ii) the diazonium salt couples with C_12_H_14_N_2_·2HCl to produce a red dye that can be detected at 540 nm.

### Enrichment culture and screening of microorganisms with the NH_4_^+^-N-degrading ability

The tailing samples obtained from the REE mines were mixed together (10 g per sample) for the screening experiment. Then, 50 g of the mixed sample was transferred into the enrichment medium. The mixtures were incubated at 28 °C and 120 rpm overnight. After that, 10 mL of the culture was injected into a fresh enrichment medium, followed by incubation at 28 °C and 120 rpm overnight. Then, the culture was subjected to separation using the LB agar plate to obtain single clones.

The clones were separately inoculated into the LB medium and were incubated at 28 °C and 180 rpm for 24 h. After that, the cells were collected by centrifugation (8000 rpm) and were suspended by sterilized normal saline to prepare a bacterial suspension with a density of approximately 10^9^ cells per milliliter (OD_600_ ≈ 1). Then, 10 mL of the bacterial suspension was mixed with 190 mL of screening medium in a 1 L flask, followed by incubation at 28 °C and 200 rpm for 48 h. The residual NH_4_^+^-N (from (NH_4_)_2_SO_4_ in the screening medium) was detected according to the method described previously (Yang et al. 2006). The screening medium without cell inoculation served as the control. The degradation rates of NH_4_^+^-N were calculated according to Eq. (1) to evaluate the degradation capabilities of microorganisms.

1 $$R = (C_{0} - C_{1} )/C_{0} \times 100\%$$

where *R*, *C*_0_ and *C*_1_ represented degradation rates, the concentration of NH_4_^+^-N in the control and the concentration of NH_4_^+^-N in the medium with cell inoculation, respectively.

### Morphological and biochemical characterization

The bacterium with the excellent NH_4_^+^-N-degrading capability was subjected to morphological observations and biochemical characterization. Optical microscopy, transmission electronic microscopy and scanning electron microscopy were adopted to analyze its morphological features according to the conventional methods (Chao et al. 2010; Deng et al. 2014, 2016; Prior and Perkins 1974). Its biochemical and physiological characteristics were analyzed according to the methods described previously (Faller and Schleifer 1981; Holt et al. 1994; Kloos et al. 1974; Lányi 1988), including motility, aerobism, Gram staining, spore formation, catalase activity, glucose fermentation, oxidase activity, nitrate reduction, starch hydrolysis, gelatin hydrolysis, indole production, Voges–Proskauer (V–P) reaction, citrate utilization, methyl red test, and production of hydrogen sulfide.

### PCR amplification of 16S rDNA and phylogenetic analysis

The bacterium with the excellent NH_4_^+^-N-degrading ability was further identified by phylogenetic analysis. Its genomic DNA was extracted according to the method described previously (Winnepenninckx et al. 1993). The 16S rDNA was amplified using universal primers 27F (5′-AGAGATTGATCCTGGCTCTG-3′) and 1492R (5′-GGTTTCCTTGTTACGACAT-3′) (Deng et al. 2014). The primers were synthesized by Sangon Biotech (Shanghai, China). The PCR reaction mixture was composed of genomic DNA (20 ng), 27F (50 μM), 1492R (50 μM), 10 × PCR buffer, 0.5 μL of DNA polymerase (5 U/L, TaKaRa, Japan), dNTPs (10 mM), MgCl_2_ (25 mM), and sterile ddH_2_O up to a volume of 50 μL. The PCR reactions were carried out on the LongGene MGL96G (Hangzhou, China). The PCR procedure was set as follows: (i) 95 °C for 5 min; (ii) 35 cycles of 95 °C for 30 s, 55 °C for 30 s, and 72 °C for 90 s; and (iii) 72 °C for 10 min. Then, the PCR product was sequenced by Sangon Biotech (Shanghai, China). The obtained 16S rDNA sequence was submitted to the GenBank database for the BLAST alignment. The MEGA 5 software (Tamura et al. 2011) was adopted to construct a phylogenetic tree using the neighbor-joining method (Li 2015).

### Optimization of NH_4_^+^-N degradation by the isolated bacterium

The effects of incubation time, carbon source, temperature, pH, C/N ratio, inoculum dose, and rotary speed on NH_4_^+^-N degradation were evaluated to determine the optimal conditions for NH_4_^+^-N degradation. (i) To evaluate the effect of incubation time on NH_4_^+^-N degradation, the isolated bacterium was inoculated (10%, v/v) into the screening medium (pH 7.0) containing NH_4_^+^-N (1 g/L), followed by incubation at 30 °C and 120 rpm. (ii) To determine the most suitable carbon source for NH_4_^+^-N degradation, the following compounds were added into the screening medium without glucose, respectively: saccharose, lactose, sodium propionate, potassium sodium tartrate, glucose, ethanol, sodium acetate, and sodium citrate. The bacteria (10%, v/v) were incubated for 48 h at 30 °C and 120 rpm. (iii) Regarding the most suitable temperature for NH_4_^+^-N degradation, the incubation temperature was set at 16, 20, 24, 28, 30, 32, 36, and 40 °C, respectively. (iv) To determine the most suitable pH for NH_4_^+^-N degradation, HCl or NaOH was adopted to adjust the initial pH of the screening medium to 5.0, 5.5, 6.0, 6.5, 7.0, 7.5, and 8.0, respectively. The bacteria were incubated at 30 °C and 120 rpm for 48 h. (v) The carbon nitrogen ratios (C/N; w/w) were set at 2:1, 4:1, 6:1, 8:1, 10:1, 12:1, and 14:1, respectively. The bacteria were incubated at 30 °C and 120 rpm for 48 h. (vi) For the optimization of inoculum dose, the bacteria were inoculated into the screening medium (pH 7.0) containing 1 g/L of NH_4_^+^-N, followed by incubation at 30 °C and 120 rpm for 48 h. The inoculum doses (v/v) of bacteria were set at 2, 5, 8, 10, 12, 15, and 18%, respectively. (vii) To determine the optimal rotary speed during incubation in an orbital shaker, the bacteria were inoculated (10%, v/v) into the medium (pH 7.0) containing 1 g/L of NH_4_^+^-N and were incubated at 30 °C for 48 h. The rotary speeds were set at 100, 120, 150, 180, and 210 rpm, respectively.

The screening medium (unless otherwise specified) was used in all the optimization experiments mentioned above. The medium without cell inoculation served as the negative control. The degradation rates of NH_4_^+^-N were calculated according to the method described above to determine the most suitable conditions for NH_4_^+^-N degradation.

Besides, an orthogonal design containing five factors and four levels was adopted to further optimize the conditions for NH_4_^+^-N degradation. The inoculum amount, temperature, pH, C/N ratio, and incubation time were respectively set at 6, 8, 10, 12%; 26, 28, 30, 32 °C; 6.0, 6.5, 7.0, 7.5; 5:1, 10:1, 15:1, 20:1; and 44, 48, 52, 56 h.

### Effect of the isolated bacterium on plant growth

Red soils for the growth of *Nepeta cataria* were baked at 120 °C for 6 h to remove the original bacteria in the soils. Ten seeds of *Nepeta cataria* were sown in the red soils (1 kg) with different concentrations of NH_4_^+^-N (500, 1000, 1500, and 2000 mg/kg, respectively). Then, a bacterial suspension of the isolated bacterium (1 mL, OD_600_ = 1) was inoculated into the red soils. The groups without bacterial inoculum served as the controls. The growth of *Nepeta cataria* in a humid environment was observed, and the plant lengths were measured at the time point of 12 days. Additionally, the concentrations of residual NH_4_^+^-N in the soils were detected every two days using the method described above.

### Growth of strain Gan-35 in the high salt medium

Strain Gan-35 was inoculated into the screening medium containing 1.0, 2.0, and 3.5% (w/v) of NaCl, respectively, followed by incubation at 28 °C and 120 rpm for 48 h. The absorbance of the culture at 523 nm was measured every 4 h. Then, a growth curve was drawn to evaluate the growth of strain Gan-35.

### Accession number

The 16S rDNA sequence of the isolated bacterium was submitted to the GenBank database under accession number KY928114.

## Results

### Contents of NH_4_^+^-N, NO_3_^−^-N and NO_2_^−^-N in the tailings of REE mines

The contents of NH_4_^+^-N in the tailings range from 483.2 to 899.4 mg/kg (Table 1), indicating that there is severe NH_4_^+^-N pollution in the tailings of REE mines. However, the concentrations of NO_3_^−^-N and NO_2_^−^-N are relatively low.

**Table 1:** Contents of NH_4_^+^-N, NO_3_^−^-N and NO_2_^−^-N in the tailings of rare-earth-element mines

Sample no.	NH_4_^+^-N (mg/kg)	NO_3_^−^-N (mg/kg)	NO_2_^−^-N (mg/kg)
1	837.8	151.0	7.9
2	713.6	69.8	33.3
3	821.2	84.8	16.8
4	766.5	135.2	25.5
5	663.6	152.3	8.3
6	728.9	174.6	150.1
7	483.2	70.6	37.9
8	648.1	241.0	51.6
9	677.7	293.4	18.0
10	899.4	37.1	23.7
11	888.4	118.1	15.9
12	563.2	135.0	66.4
13	587.8	56.2	45.6
14	890.2	70.3	24.5
15	653.6	87.8	39.5
16	770.9	121.1	229.2

### Screening of NH_4_^+^-N-degrading strains

**Fig. 2:**
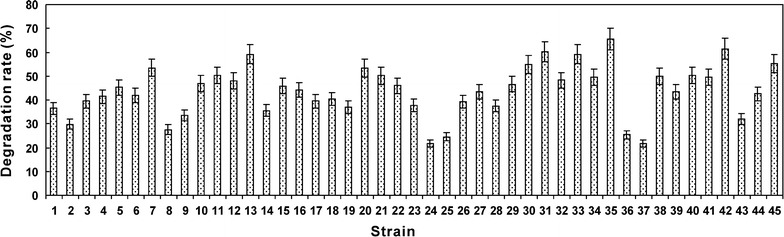
Strains with the NH_4_^+^-N-degrading ability. The degradation rates were detected after incubation for 2 days. The detections were performed in triplicate, and the results were presented as mean ± standard deviation

The screening experiment showed that 45 strains with the NH_4_^+^-N-degrading ability were obtained from the tailings of REE mines. The degradation rates against NH_4_^+^-N (1 g/L) ranged from 21.6 to 65.6% (Fig. 2) after incubation for two days. Strain Gan-35 exhibited the highest degradation rate and was selected for further investigation. Additionally, the concentrations of NO_3_^−^-N and NO_2_^−^-N in the screening medium during NH_4_^+^-N degradation were measured, and the results showed that their contents were very low (Additional file 1: Tables S1, S2). Only 0.95% and 0.06% of NH_4_^+^-N were transformed into NO_3_^−^-N and NO_2_^−^-N, respectively, suggesting that strain Gan-35 may not cause secondary pollution during NH_4_^+^-N degradation.

### Identification of strain Gan-35

**Fig. 3:**
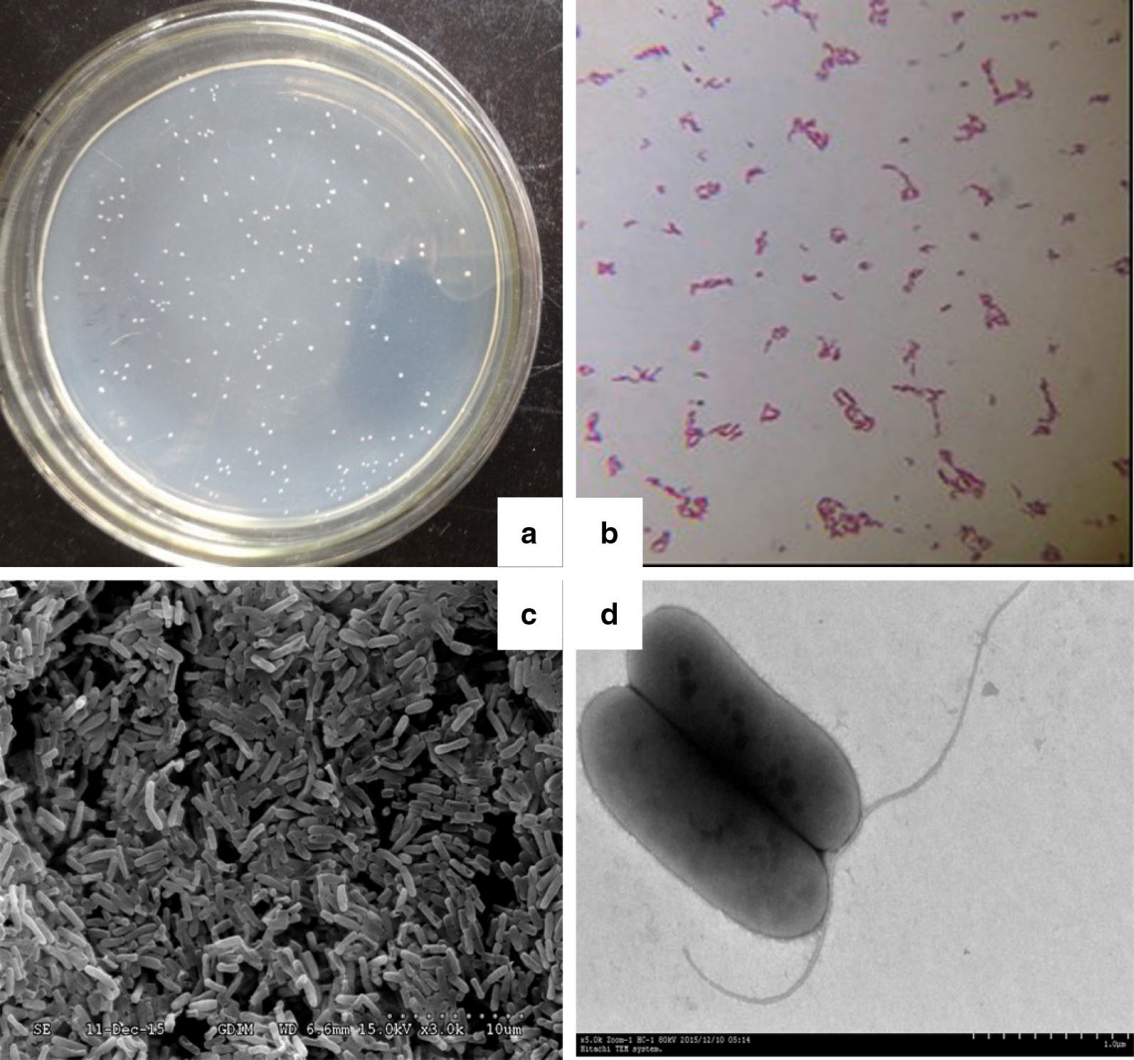
Morphological characteristics of strain Gan-35. **a** Colonies of strain Gan-35 on the LB agar plate; **b** a photograph of Gram staining (20 × 100); **c** a photograph of scanning electron microscopy (×15,000); and **d** a photograph of transmission electronic microscopy (×5000)

The colony of strain Gan-35 was shown to be smooth-faced, white-colored, and circular with a tidy margin (Fig. 3a). This strain is a Gram-negative bacterium (Fig. 3b). The results of scanning electron microscopy (×15,000) and transmission electronic microscopy (×5000) showed that strain Gan-35 was a rod-shaped cell with a size of (0.5–0.8) μm × (1.0–2.1) μm and contained flagella on the cell surface (Fig. 3c, d). The biochemical and physiological characteristics of strain Gan-35 were shown in Table 2. Briefly, strain Gan-35 is an aerobic and motile bacterium with the activities of catalase and oxidase. This strain is positive for nitrate reduction, starch hydrolysis, gelatin hydrolysis, and Voges–Proskauer reaction. It is negative for indole production, citrate utilization, spore formation, and production of hydrogen sulfide. In summary, the characteristics of strain Gan-35 are consistent with the descriptions of *Bergey’s Manual of Determinative for Bacteriology* (Holt et al. 1994) regarding *Burkholderia*.

**Table 2:** Morphological, physiological and biochemical characteristics of strain Gan-35

Characteristics	Strain Gan-35
Morphology	Rod shaped
Colony color	White
Gram staining	−
Motility	+
Aerobism	+
Spore formation	−
Oxidase activity	+
Catalase activity	+
Glucose fermentation	−
Nitrate reduction	+
Starch hydrolysis	+
Gelatin hydrolysis	+
Methyl red test	−
Citrate utilization	−
Indole production	−
Voges–Proskauer reaction	+
Production of hydrogen sulfide	−

−, negative; +, positive

**Fig. 4:**
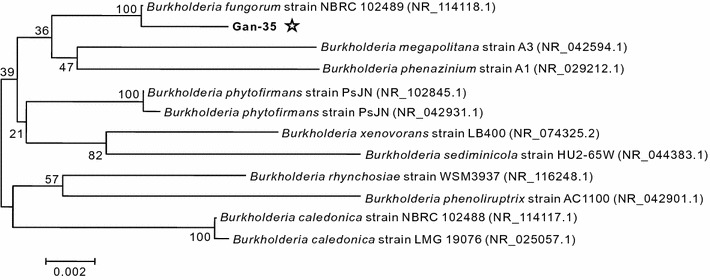
Phylogenetic analysis using 16S rDNA sequences. The accession numbers of the 16S rDNA sequences are presented in the parentheses. Strain Gan-35 is indicated by a pentagram

DNA sequencing showed that the obtained 16S rDNA sequence (1425 bp) of strain Gan-35 was highly homologous (99% identity) to that of *Burkholderia* strains. A phylogenetic tree (Fig. 4) was constructed according to the similar 16S rDNA sequences using the neighbor-joining method (Li 2015). The result showed that strain Gan-35 was most closely related to *Burkholderia fungorum* strain NBRC 102489 (accession number: NR_114118.1).

Based on the morphological, biochemical and physiological properties and phylogenetic analysis of 16S rDNA sequences, strain Gan-35 was identified as *Burkholderia fungorum*. Strain Gan-35 was deposited in the China Center for Type Culture Collection with the preservation number of “CCTCC AB 2017087”.

### Optimization of the conditions for NH_4_^+^-N degradation

**Fig. 5:**
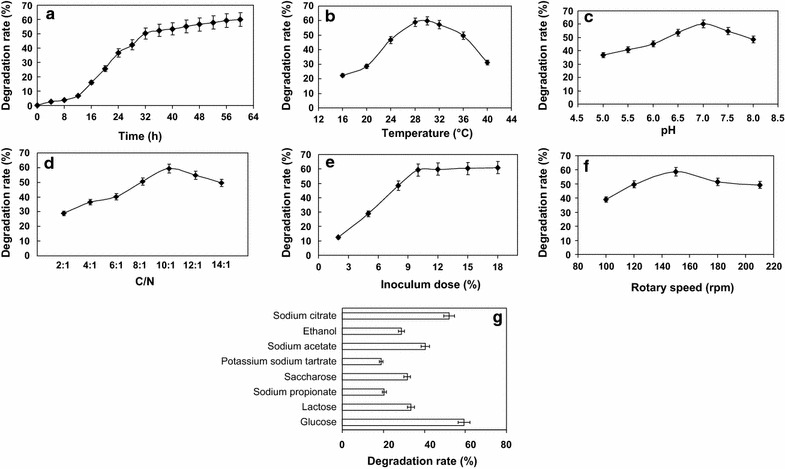
Optimization of the conditions for NH_4_^+^-N degradation. The optimized conditions included incubation time (**a**), temperature (**b**), pH (**c**), C/N ratio (**d**), inoculum dose (**e**), rotary speed (**f**), and carbon source (**g**). The degradation tests were performed in triplicate, and the results were shown as mean ± standard deviation

The effects of incubation time, carbon source, temperature, pH, C/N ratio, inoculum dose, and rotary speed on the degradation of NH_4_^+^-N by strain Gan-35 were investigated in this study. It can be seen from Fig. 5a that the degradation rates of NH_4_^+^-N increase rapidly after the time point of 12 h, and after 32 h the increase is less obvious. Under the conditions of 16–30 °C, the degradation rates increase with the enhancement of temperatures. Strain Gan-35 can degrade 59.8% of NH_4_^+^-N at 30 °C, and the degradation rates decrease when the temperatures are higher (Fig. 5b). Strain Gan-35 exhibits the highest degradation rates (60.1%) against NH_4_^+^-N at an initial pH value of 7.0 (Fig. 5c). The degradation rates decrease when the pH values are lower or higher, suggesting that a neutral environment is more suitable for the NH_4_^+^-N degradation by strain Gan-35. As shown in Fig. 5d, the highest degradation rate (59.3%) was obtained when the C/N ratio was set at 10:1. It also suggests that a greater C/N ratio does not necessarily increase the degradation rates. The degradation rates increase significantly when the inoculum doses are at the interval of 2–10% (Fig. 5e). The changes of degradation rates are not obvious if the inoculum doses are greater than 10%, which may be due to that the nutrients are relatively deficient when the inoculum doses are greater. A higher degradation rate was observed when the rotary speed of an orbital shaker was set at 150 rpm (Fig. 5f), suggesting that moderate dissolved oxygen is needed for the efficient degradation of NH_4_^+^-N. Besides, strain Gan-35 exhibits the highest degradation rate of NH_4_^+^-N (59.4%) using glucose (followed by sodium citrate and sodium acetate) as the sole carbon source (Fig. 5g).

An orthogonal design involving five factors and four levels was performed in this study. Range analysis and variance analysis were adopted to determine the optimal conditions for NH_4_^+^-N degradation, and the results were shown in Tables 3 and 4. The variance analysis indicates that there are differences among inoculum dose, temperature, pH, C/N ratio, and incubation time. Their effects on NH_4_^+^-N degradation are significant (*p* < 0.05) and are in the following order: temperature >pH >C/N ratio >incubation time >inoculum dose. According to the range analysis, the optimal conditions for NH_4_^+^-N degradation in an indoor laboratory was determined as follows: pH value, 7.5; inoculum dose, 10%; incubation time, 44 h; temperature, 30 °C; and C/N ratio, 15:1. A mean degradation rate of 68.6% was obtained under the above optimal conditions. The optimization of NH_4_^+^-N degradation by strain Gan-35 contributes to designing effective methods for bioremediation of NH_4_^+^-N-polluted environments, such as producing effective microbial inocula for bioaugmentation.

**Table 3:** Orthogonal design for NH_4_^+^-N degradation

No.	Inoculum dose (%)	Temperature (°C)	pH value	C/N ratio	Incubation time (h)	Degradation rate (%)
1	6	26	6.0	5:1	44	49.7 ± 2.3
2	6	28	6.5	10:1	48	50.8 ± 1.9
3	6	30	7.0	15:1	52	66.1 ± 1.2
4	6	32	7.5	20:1	56	59.4 ± 1.5
5	8	26	6.5	15:1	56	51.2 ± 2.2
6	8	28	6.0	20:1	52	53.7 ± 2.8
7	8	30	7.5	5:1	48	66.8 ± 2.3
8	8	32	7.0	10:1	44	51.5 ± 2.0
9	10	32	7.0	20:1	48	52.4 ± 1.1
10	10	30	7.5	15:1	44	68.6 ± 1.4
11	10	28	6.0	10:1	56	65.2 ± 1.6
12	10	26	6.5	5:1	52	51.2 ± 2.5
13	12	26	7.5	10:1	52	52.1 ± 1.4
14	12	28	7.0	5:1	56	51.8 ± 2.8
15	12	30	6.5	20:1	44	65.1 ± 2.2
16	12	32	6.0	15:1	48	53.1 ± 2.4
$\overline{{k_{1} }}$	56.500	51.350	55.425	54.875	59.225	
$\overline{{k_{2} }}$	55.800	55.975	55.325	54.900	55.775	
$\overline{{k_{3} }}$	59.100	66.550	55.450	59.500	55.775	
$\overline{{k_{4} }}$	56.275	53.800	61.475	58.400	56.900	
Range	3.300	15.200	6.150	4.625	3.450	

The degradation rates are presented as mean ± standard deviation

**Table 4:** Variance analysis for the conditions of NH_4_^+^-N degradation

Factor	Square of deviance	d*f*	*F* ratio	*F* critical value	Significance
Inoculum dose	26.397	3	0.170	3.290	
Temperature	537.557	3	3.469	3.290	*
pH	100.752	3	0.715	3.290	
C/N	68.437	3	0.442	3.290	
Incubation time	31.742	3	0.205	3.290	
Error	774.88	15			

* Significantly different (*p* < 0.05)

### Effects of strain Gan-35 on plant growth and its tolerance to the high salinity

**Fig. 6:**
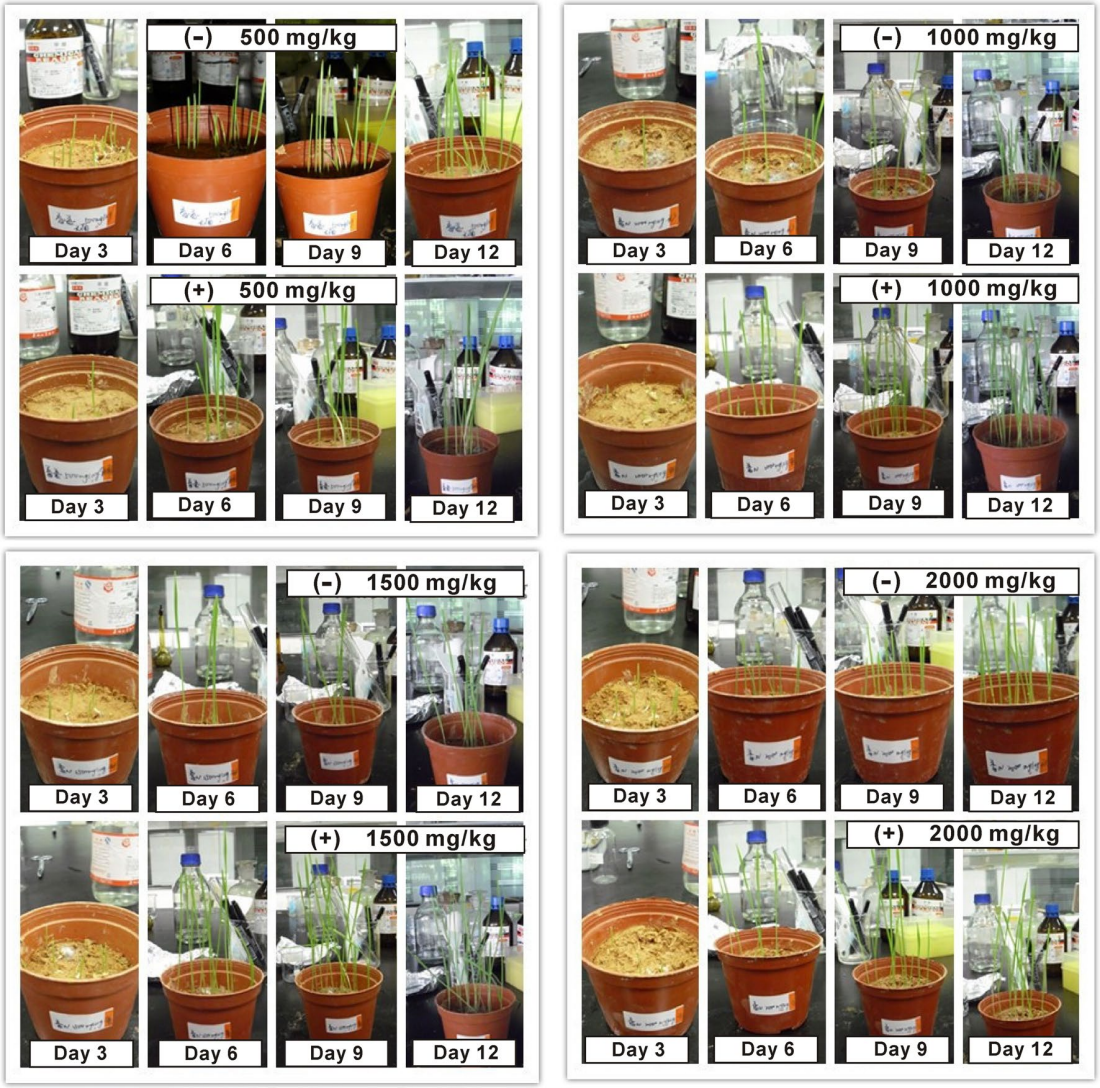
The promotion effect of strain Gan-35 on the growth of *Nepeta cataria*. *Nepeta cataria* was grown in the red soils containing NH_4_^+^-N at the concentrations of 500, 1000, 1500, and 2000 mg/kg, respectively. (−), without Gan-35; (+), with Gan-35

**Fig. 7:**
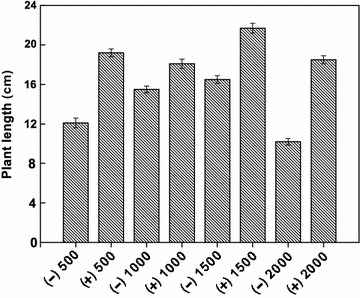
Lengths of *Nepeta cataria* during growth in the red soils containing NH_4_^+^-N. (−), without Gan-35; (+), with Gan-35. The numbers on the *horizontal axis* are the concentrations of NH_4_^+^-N (mg/kg)

*Burkholderia* is a genus rich in nitrogen-fixing and phosphate-solubilizing strains that have been isolated from various plant systems. The functions of phosphate-solubilizing bacteria in agriculture have been well documented, including enhancements in growth, yield and disease-resistance of crops (Ghosh et al. 2016). The effects of *Burkholderia fungorum* Gan-35 on plant growth were investigated in this study. As shown in Fig. 6, *Nepeta cataria* with Gan-35 inoculum grew obviously better than that without inoculation. The average plant lengths of the former are significantly greater than the latter (Fig. 7). Besides, the NH_4_^+^-N contents in the red soils decreased obviously along with the time extension in the experimental groups with Gan-35 inoculum, and the degradation rates of NH_4_^+^-N were between 43.37 and 51.42% (Table 5). Nevertheless, the decrease of NH_4_^+^-N contents in the controls was not obvious, and the degradation rates were between 6.12 and 10.30%. Thus, it is suggested that the degradation of NH_4_^+^-N performed by strain Gan-35 can relieve the growth inhibition effect caused by the high concentrations of NH_4_^+^-N. In other words, Gan-35 inoculation in the soils with NH_4_^+^-N pollution contributes to the growth of *Nepeta cataria*.

**Table 5:** NH_4_^+^-N contents in the red soils with time extension

Time (d)	Concentration of NH_4_^+^-N (mg/kg)							
	500 (+)	500 (−)	1000 (+)	1000 (−)	1500 (+)	1500 (−)	2000 (+)	2000 (−)
0	547.92	539.69	1093.27	1121.02	1605.42	1589.48	2128.18	2131.26
2	439.76	530.27	903.55	1103.54	1481.53	1556.12	1803.37	2090.11
4	372.84	521.89	784.51	1096.26	1377.28	1539.47	1657.23	2049.15
6	340.64	510.68	700.36	1088.47	1236.01	1521.91	1492.84	2017.34
8	306.61	506.12	646.78	1071.22	1099.32	1498.98	1375.27	1995.77
10	282.79	500.09	598.32	1061.32	995.50	1482.42	1284.41	1941.36
12	266.17	496.46	540.16	1052.43	885.94	1473.22	1205.21	1911.69
Degradation rate (%)	51.42	8.01	50.59	6.12	44.82	7.31	43.37	10.30

The concentrations of NH_4_^+^-N are presented as the average values of two determinations

d, day; (−), without Gan-35; (+), with Gan-35

**Fig. 8:**
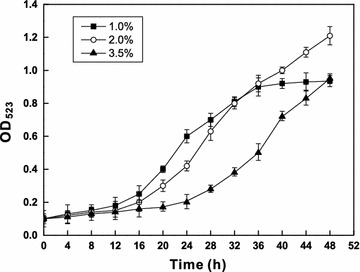
Growth curve of strain Gan-35 during growth in the high salt medium. The concentrations of NaCl in the medium were set at 1.0, 2.0, and 3.5% (w/v), respectively. The absorbance of the culture at 523 nm was measured every 4 h

The growth of strain Gan-35 in the high salt medium showed that this bacterium entered a rapid growth phase after the time points of 12 or 20 h when the concentrations of NaCl were set at 1.0, 2.0, and 3.5%, respectively (Fig. 8). The bacteria were still in the rapid growth period at the time point of 48 h in the media with 2.0 or 3.5% of NaCl, suggesting that strain Gan-35 exhibits the tolerance to the high salinity, which contributes to the bioremediation in hyperhaline NH_4_^+^-N-polluted environments and the removal of NH_4_^+^-N in aquatic environments in marine farms.

## Discussion

The detection of NH_4_^+^-N contents in the tailings of REE mines suggests that the NH_4_^+^-N pollution in these areas is severe. The studied samples were collected at the depth of 10–15 cm in the tailings. We infer that the contents of NH_4_^+^-N may be higher at deeper positions due to the cumulative effect, and that a part of NH_4_^+^-N in the surface tailings has been transferred into aquatic environments by rain wash. The severe NH_4_^+^-N pollution has induced many negative effects on the surrounding ecosystems and human health (Åström 2001). Thus, reducing the NH_4_^+^-N pollution and remediating its contaminated environment are imperative.

So far, some functions of *Burkholderia* strains have been demonstrated, such as biological control, promoting plant growth, bioremediation, and producing various active metabolites, including phenazine, pyroace, and monoterpenoid alkaloids (Coenye et al. 2001). Besides, *Burkholderia* strains had been applied as biological insecticides, biological bactericides and decomposition of toxic substances. To our knowledge, the NH_4_^+^-N-degrading ability of *Burkholderia* sp. has not been discovered before. Thus, this study has reported a *Burkholderia* strain with the NH_4_^+^-N-degrading capability for the first time. The obtained results provide a new insight into the promising applications of *Burkholderia* strains in terms of bioremediation of NH_4_^+^-N-polluted environments. This is also the first report on the isolation and characterization of a bacterium with the NH_4_^+^-N-degrading ability from the tailings of REE mines, laying the foundation for the bioremediation of these areas.

In situ bioremediation using indigenous microorganisms is an effective method to eliminate pollutants (Lin et al. 2012). In some cases, indigenous microorganisms with the pollutant-degrading ability may be better adapted for bioremediation. Since strain Gan-35 is an indigenous bacterium isolated from the NH_4_^+^-N-polluted tailings of REE mines and exhibits the ability for (i) the degradation of NH_4_^+^-N at high concentrations, (ii) promoting plant growth, and (iii) resistance to high salinity, it is plausible that strain Gan-35 can be applied in the bioremediation of these areas. Unraveling the mechanisms for NH_4_^+^-N degradation in strain Gan-35 and extensive field studies in the future, such as revealing the relative abundance of strain Gan-35 in the tailings of REE mines, contribute to realizing its practical applications for bioremediation.

In summary, a bacterium with the NH_4_^+^-N-degrading capability has successfully been isolated from the tailings of REE mines. This strain is identified as *Burkholderia fungorum* Gan-35 on the basis of phylogenetic analysis and its phenotypic characteristics. This is the first study to report a *Burkholderia* strain with the NH_4_^+^-N-degrading ability. This is also the first research on the screening of a bacterium with the NH_4_^+^-N-degrading ability from the tailings of REE mines. The optimal conditions for NH_4_^+^-N degradation in strain Gan-35 have been determined, which provides valuable information for designing effective methods for its applications in bioremediation. Besides, strain Gan-35 exhibits the abilities of promoting plant growth and resistance to high salinities. This work contributes to developing a cost-effective and eco-friendly method for bioremediation of the tailings of REE mines contaminated by NH_4_^+^-N.

## Additional file

**Additional file 1.** Tables S1, S2.

## Abbreviations

REE: rare earth element
NH_4_^+^-N: ammonia nitrogen
h: hour
NO_2_^−^-N: nitrite nitrogen
LB: Luria–Bertani
rpm: revolutions per minute
PCR: polymerase chain reaction
OD: optical density
