# Supplementary material for: Isolation and characterization of Burkholderia fungorum Gan-35 with the outstanding ammonia nitrogen-degrading ability from the tailings of rare-earth-element mines in southern Jiangxi, China
Source: AMB Express. 2017 Jun 26;7:140. doi: 10.1186/s13568-017-0434-x (PMC5484655; doi:10.1186/s13568-017-0434-x)
Supplement: Supplementary file 1 — Additional file 1. Tables S1, S2. [file 13568_2017_434_MOESM1_ESM.doc]

**Additional Files**

**Journal name:** AMB Express

**Manuscript title:** Isolation and characterization of *Burkholderia* *fungorum* Gan-35 with the outstanding ammonia nitrogen-degrading ability from the tailings of rare-earth-element mines in southern Jiangxi, China

**Authors:** Ai-Juan Feng^1,2^, Xi Xiao^2^, Cong-Cong Ye^2^, Xiao-Ming Xu^2^, Qing Zhu^2^,

Jian-Ping Yuan^2^, Yue-Hui Hong^2^,*, Jiang-Hai Wang^2^,*

^1^ School of Life Sciences, Sun Yat-Sen University, Guangzhou 510275, People’s Republic of China

^2^ Guangdong Provincial Key Laboratory of Marine Resources and Coastal Engineering/ South China Sea Bioresource Exploitation and Utilization Collaborative Innovation Center, School of Marine Sciences, Sun Yat-Sen University, Guangzhou 510006, People’s Republic of China

## *Correspondence: wangjhai@mail.sysu.edu.cn (Jiang-Hai Wang); yuehuihong@126.com (Yue-Hui Hong). Tel.: +86-20-39332212; Fax: +86-20-39332213.

**Email addresses of other authors:**

9606707@qq.com (Ai-Juan Feng);

xiaox46@mail2.sysu.edu.cn (Xi Xiao);

1571309420@qq.com (Cong-Cong Ye);

xxm8302@126.com (Xiao-Ming Xu);

463685446@qq.com (Qing Zhu);

yuanjp@mail.sysu.edu.cn (Jian-Ping Yuan)

**Table S1 Contents of NO_3_^−^-N in the medium during NH_4_^+^**-N degradation

| Degradation time (h) | Contents of NO_3_^−^-N (mg/L) |
| --- | --- |
| 0 | 0.0173 |
| 2 | 0.0443 |
| 4 | 0.0443 |
| 6 | 0.1039 |
| 8 | 0.1443 |
| 10 | 0.1828 |
| 12 | 0.2213 |
| 14 | 0.2762 |
| 16 | 0.2929 |

**Table S2 Contents of NO_2_**^−^**-N in the medium during NH_4_^+^-N degradation**

| Degradation time (h) | Contents of NO_2_^−^-N (mg/L) |
| --- | --- |
| 0 | 0 |
| 2 | 0 |
| 4 | 0.0003 |
| 6 | 0.0011 |
| 8 | 0.0020 |
| 10 | 0.0097 |
| 12 | 0.0160 |
| 14 | 0.0171 |
| 16 | 0.0185 |
